# Proteolytic Volatile Profile and Electrophoretic Analysis of Casein Composition in Milk and Cheese Derived from Mironutrient-Fed Cows

**DOI:** 10.3390/molecules25092249

**Published:** 2020-05-10

**Authors:** Andrea Ianni, Francesca Bennato, Camillo Martino, Lisa Grotta, Nicola Franceschini, Giuseppe Martino

**Affiliations:** 1 Faculty of BioScience and Technology for Food, Agriculture and Environment, University of Teramo, Via Renato Balzarini 1, 64100 Teramo, Italy; aianni@unite.it (A.I.); fbennato@unite.it (F.B.); lgrotta@unite.it (L.G.); 2 Istituto Zooprofilattico Sperimentale dell’Abruzzo e del Molise “G. Caporale”, Via Campo Boario 37, 64100 Teramo, Italy; c.martino@izs.it; 3 Department of Biotechnological and Applied Clinical Sciences, University of L’Aquila, Via Vetoio 1, 67100 L’Aquila, Italy; nicola.franceschini@univaq.it

**Keywords:** proteolysis, microelement, dairy cow, caciocavallo cheese, casein, volatile compound

## Abstract

The aim of the study was to evaluate the proteolytic process in Caciocavallo cheese obtained from Friesian cows fed zinc, selenium, and iodine supplementation. Thirty-six Friesian cows, balanced for parity, milk production, and days in milk, were randomly assigned to four groups. The control group (CG) was fed with a conventional feeding strategy, while the three remaining groups received a diet enriched with three different trace elements, respectively zinc (ZG), selenium (SG), and iodine (IG). At the end of the experimental period, samples of milk were collected and used to produce Caciocavallo cheese from each experimental group. Cheese samples were then analyzed after 7 and 120 days from the cheese making in order to obtain information on chemical composition and extent of the proteolytic process, evaluated through the electrophoretic analysis of caseins and the determination of volatiles profile. Both milk and cheese samples were richer in the amount of the microelement respectively used for the integration of the cattle’s diet. The zymographic approach was helpful in evaluating, in milk, the proteolytic function performed by endogenous metalloenzymes specifically able to degrade gelatin and casein; this evaluation did not highlight significant differences among the analyzed samples. In cheese, the electrophoretic analysis in reducing and denaturing condition showed the marked ability of β-casein to resist the proteolytic action during ripening, whereas the dietary selenium supplementation was shown to perform a protective action against the degradation of S1 and S2 isoforms of α-casein. The analysis of the volatile profile evidenced the presence of compounds associated with proteolysis of phenylalanine and leucine. This approach showed that selenium was able to negatively influence the biochemical processes that lead to the formation of 3-methyl butanol, although the identification of the specific mechanism needs further investigation.

## 1. Introduction

High-yielding animals require feeding strategies that guarantee the right contribution of all the necessary microelements, such as zinc, manganese, copper, cobalt, iodine, and selenium. Dietary microelements deficiency in livestock commonly leads to a wide range of disorders especially associated to growth depression, inefficient feed utilization, lower production performance, and depressed immunocompetence that may increase animals’ susceptibility to infectious diseases [1].

Zinc is a ubiquitous element in cells and represents an essential component of several metalloenzymes [2] and transcription factors, with relevant roles in the metabolism of essential nutrients in animals. Zinc is not stored in the animal body; therefore, a constant dietary supply is necessary to avoid the onset of a wide range of pathological conditions, such as skin parakeratosis, reduction or cessation of growth, general debility, lethargy, and increased susceptibility to infection [3]. Selenium is involved in numerous biological mechanisms, including cellular response to oxidative stress, redox signaling, cellular differentiation, immune response, and protein folding [4]. Selenium was also reported to improve rumen fermentation, milk yields, and feed digestion in Holstein dairy cows [5]. Iodine is the main component of the thyroid hormones and when its requirement is not satisfied, a reduced functionality of the thyroid gland could occur (hypothyroidism) with consequences for proper mental development, body growth, and decreased fertility. In animal husbandry, iodine supplementation is needed because the native iodine content of plant straight feed-stuffs is low; moreover, the increasing use of rapeseed meal in livestock diets is associated with the intake of glucosinolates, which are known to be iodine antagonists, inhibiting the activity of sodium iodide symporter [6].

Different studies have been carried out in order to evaluate the effect of dietary microelements intake on ruminants’ metabolism [7,8,9] and chemical-nutritional quality of dairy products [10,11,12], but the topic concerning the microelements’ influence on ripened cheese flavor has received less attention. Conversion of lactose and citrate, lipolysis, and proteolysis represent the main chemical processes involved in the development of aroma in dairy products. Among these processes, proteolysis of caseins is an important biochemical pathway responsible for the formation of flavor and texture in hard- and semi hard-type cheeses [13]. Proteolysis in cheese can be divided into the primary and the secondary phase. Primary proteolysis is performed by rennet, native milk enzymes and induces degradation of caseins into large, well-defined polypeptides. Further proteolytic processes operated by starter and nonstarter bacteria during ripening contribute to secondary proteolysis, which cause formation of small polypeptides and free amino acids responsible for cheese aroma and taste [14]. Branched chain amino acids (leucine, isoleucine, and valine), aromatic amino acids (phenylalanine, tyrosine, and tryptophan), and methionine are thought to be the precursors of important volatile compounds in dairy products [15]. Therefore, the aim of the present study was to investigate the effect of dietary microelements’ intake on the development of proteolysis in fresh and ripened dairy products obtained from lactating dairy cows. Specifically, the study was conducted on Caciocavallo cheese, a dairy product of bovine origin, which is generally subjected to seasoning for fairly long intervals of time compared to other products, and which is therefore more exposed to both lipolytic and proteolytic processes. The study in any case did not concern only the cheese but was also extended to the milk used for cheesemaking, in order to verify the actual enrichment with the microelements respectively used for dietary supplementation and also to evaluate the presence and the activity of native milk metalloenzymes, which exploit these microelements, especially zinc, as cofactors.

## 2. Results

### 2.1. Microelements Quantification in Milk and Cheese

At the end of the 56 days of the trial, milk samples obtained from each experimental group (zinc (ZG), selenium (SG), and iodine (IG)) in the feeding strategy were found to be effective in inducing an enrichment of the microelement respectively used for the dietary supplementation (Table 1).

The finding concerning the enrichment with the microelements used for the dietary supplementation was also found in samples of Caciocavallo cheese, both fresh (T_7_) and after 120 days of repining (T_120_). The results concerning the quantification performed on individual samples for the two ripening times is shown in Table 2.

### 2.2. Zymographic Evaluation of Gelatinolytic and Caseinolytic Activity in Milk

Enzymatic activities able to induce the degradation of gelatin and casein in milk samples have been evaluated using a zymographic approach.

**Figure 1:**
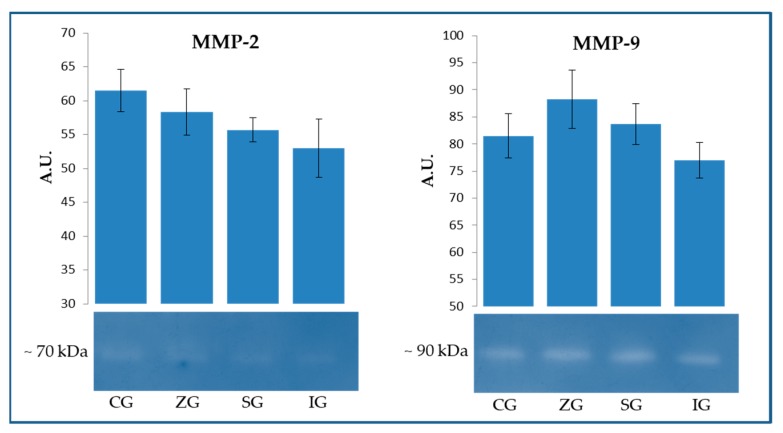
Gelatin-zymography on milk samples obtained from lactating dairy cows fed control diet (CG) and control diet supplemented with zinc (ZG), selenium (SG), and iodine (IG). Analysis was performed in order to obtain information on the enzymatic activities associated to matrix metalloproteinase 2 (MMP-2) and matrix metalloproteinase 9 (MMP-9). The ImageJ software was used to perform the quantitative analysis of visualized spots. Data are reported as mean values expressed in arbitrary unit (A.U.) ± standard deviation.

The gelatin-zymography (Figure 1) was helpful in highlighting the enzymatic activity closely associated with the two major gelatinases: matrix metalloproteinase 2 (MMP-2) and matrix metalloproteinase 9 (MMP-9).

With specific regard to MMP-2, no significant variations were detected (*p* > 0.05), although the quantitative analysis of the spots highlighted a tendency in the samples from the experimental groups (ZG, SG, and IG) to degrade gelatin with less efficacy. In the case of MMP-9, all samples showed greater activity than that found for MMP-2. However, similarly to what was observed for MMP-2, no significant differences between the various samples were identified (*p* > 0.05). The only noteworthy phenomenon concerns the slight tendency of the ZG sample to degrade the substrate more effectively.

**Figure 2:**
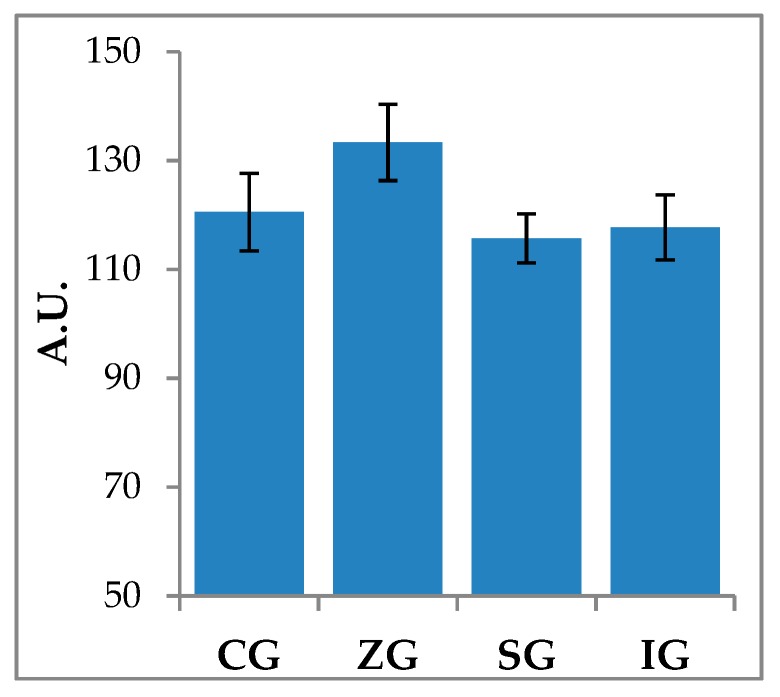
Casein-zymography on milk samples obtained from lactating dairy cows fed control diet (CG) and control diet supplemented with zinc (ZG), selenium (SG), and iodine (IG). The ImageJ software was used to perform the quantitative analysis. Data are reported as mean values expressed in arbitrary unit (A.U.) ± standard deviation.

Total caseinolytic activity was assessed through casein-zymography. The data shown in Figure 2 showed a picture quite similar to that observed for MMP-9 with the ZG milk sample, which seemed to have a greater ability to degrade the substrate, although this difference compared to the control (CG) and to the other experimental samples (SG and IG) was not significant (*p* > 0.05).

### 2.3. Caseins Separation by Sodium Dodecyl Sulfate Polyacrylamide Gel Electrophoresis (SDS-PAGE)

**Figure 3:**
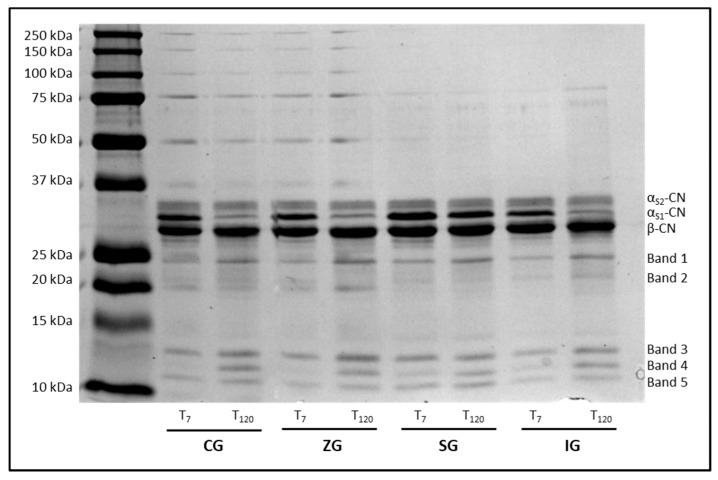
Sodium dodecyl sulfate polyacrylamide gel electrophoresis (SDS-PAGE) pattern of caseins and peptides resulting from protein degradation in fresh (T_7_) and 120-days ripened (T_120_) cheese samples obtained from lactating dairy cows fed control diet (CG) and control diet supplemented with zinc (ZG), selenium (SG), and iodine (IG) deviation.

The sodium dodecyl sulfate polyacrylamide gel electrophoresis (SDS-PAGE) was performed with the aim of monitoring the degradation of caseins by the rennet and indigenous milk enzymes. As showed in Figure 3, cheese proteins have been separated into three major casein components (αS1-CN, αS2-CN, and β-CN).

Under the applied experimental conditions, the protein profile of both fresh (T_7_) and ripened (T_120_) cheese showed a major β-CN band and less intensive bands corresponding to αS1-CN and αS2-CN. In all samples, five low molecular weight peptides (from 25 kDa to 10 kDa) were also identified as proteolysis products (Table 3). Dietary supplementation with zinc and iodine did not generate significant changes compared to the CG samples both at T_7_ and at T_120_, while selenium influenced the proteolytic process, partly protecting αS2-CN, as evidenced by the lack of significant differences in the proteolysis products corresponding to bands 2, 3, and 4 in SG samples (*p* > 0.05).

### 2.4. Identification of Volatile Compounds in Fresh and Ripened Cheese

Volatile compounds resulting from secondary proteolysis during ripening were identified in all the analyzed samples (Table 4). Two volatile compounds, phenylacetaldehyde and 2-phenylethyl alcohol, derived from phenylalanine catabolism were identified, while only the 3-methyl-1-butanol was identified as a derivative of leucine degradation. 

In both T_7_ and T_120_ samples no significant variations were found in the phenylacetaldehyde content (*p* > 0.05). In the case of 2-phenylethyl alcohol, dietary supplementation with zinc, selenium, and iodine, seems to have led to a significant reduction of this compound in T_7_ samples (6499 AU in CG vs. 3125, 1364, and 3037 AU in ZG, SG, and IG, respectively; *p* < 0.01). In ripened cheese, the phenomenon was confirmed only in ZG samples, while an increase of 2-phenylethyl alcohol was detected in IG. The only identified compound deriving from the leucine degradation was 3-methyl butanol, which tends to be synthesized in the various cheese samples with a comparable pattern both after 7 and 120 days of ripening. Specifically, no significant differences were observed between CG, ZG, and IG samples, while both at T_7_ and T_120_, the SG samples were characterized by a lower concentration of this compound (*p* < 0.01 at T_7_ and *p* < 0.05 at T_120_).

## 3. Discussion

Preventive analysis performed to determine the chemical composition of cheese samples obtained from the various experimental groups did not show significant changes both in relation to the feeding strategy (CG, ZG, SG, and IG) and in relation to the ripening time (T_7_ and T_120_). In particular, there were no differences in the protein content (Supplementary Table S1), testifying that the proteolytic process took place in the presence of equal substrate concentrations among the analyzed cheese samples. This finding is in complete agreement with what has been observed in other studies, which tested the dietary supplementation with essential trace elements in dairy cows [10,16]. With regards to the dosage of zinc, selenium, and iodine in milk and cheese samples, a significant increase of the micronutrient respectively used for the integration of the animal diet was highlighted. Limited to selenium, this result is in agreement with several studies [17], while in the case of zinc and iodine, there are discrepancies with what was previously reported. Pechová et al. [18] evidenced the inability of dietary zinc supplementation to influence its amount in bovine milk and cheese; these authors discussed such phenomenon advancing the hypothesis of an impaired incidence of rumen acidosis in the herd before the start of the experiment. With regard to iodine, Moschini et al. [19] indicated this microelement suitable for milk fortification through feed supplementation but its poor ability to interact with protein structures seems to compromise its transfer to cheese. Given the relevance of zinc, selenium, and iodine in human biochemical mechanisms, their general enrichment in milk and dairy products at the concentrations detected in this study should represent a positive aspect. However, it must be taken into account that an excess of these trace elements can constitute a technological risk factor for dairy products, both for the commercial image of the products and, above all, for consumers’ health. This consideration assumes particular value especially in the case of nonessential or toxic metals, such as lead and cadmium, that even in low concentrations are responsible for metabolic disorders with extremely serious consequences [20].

The milk enrichment with the microelements respectively used for dietary supplementation made it necessary to verify the possibility that this event could influence the function of endogenous enzymes. In particular, attention was focused on the activity of matrix metalloproteinases (MMPs), which, following cheesemaking, can be incorporated into the dairy matrix, actively participating in the proteolytic events that characterize the ripening process, especially in the initial stages. MMPs represent a family of calcium-dependent endopeptidases, with a catalytic domain containing a zinc ion coordinated by three histidines. These enzymes are involved in the physiological degradation of the extracellular matrix (ECM) in mammals, a fundamental process for tissue development, morphogenesis, remodeling, and repair. Based on their specificity for the substrate, MMPs are divided into four main subgroups: gelatinases, collagenases, stromelysins, and membrane type MMPs (MT-MMPs) [21]. In this study, the activity of gelatinases MMP-2 (Gelatinase A) and MMP-9 (Gelatinase B) have been specifically evaluated; furthermore, the caseinolytic potential of milk samples was also assayed, since casein can be hydrolyzed by different metalloenzymes, such as MMP-1 (Collagenase-1) and MMP-3 (Stromelysin-1) [22]. Although MMPs are constitutively present in all tissues, their release in milk can be partly influenced by the health status of the mammary gland. As previously reported, the inflammatory process during mastitis results in the release of a wide range of proteolytic enzymes, which are mainly secreted by polymorphonuclear cells recruited into the mammary gland from blood circulation [23]. 

The dairy cows involved in this study maintained good health conditions for the entire duration of the trial, and the preliminary evaluations carried out on milk used for cheesemaking showed particularly low values associated with somatic cells count (SCC) and without significant changes in the data associated with the total bacterial count (data not shown). The zymographic approach did not evidence significant variations both for gelatinolytic (MMP-2 and MMP-9) and caseinolytic activities. Because these enzymes depend on the presence of zinc, an increase in the substrate hydrolysis capacity in ZG samples would have been expected. The justification for this evidence could be sought in the fact that dietary supplementation was performed by using zinc oxide (ZnO), which showed, in alternative research fields, the ability to even block the activity of MMP-9 [24]. It is therefore presumable that this organic form of zinc is difficult to use by this family of enzymes, which therefore do not undergo an improvement in the hydrolysis kinetics of the respective substrates.

Regarding the analysis of primary proteolysis in cheese, the SDS-PAGE has proved to be particularly useful in monitoring the degradation of caseins by the rennet and indigenous milk enzymes. Cheese proteins have been separated into three major casein components (αS1-CN, αS2-CN, and β-CN) in all the analyzed samples, with molecular weights and electrophoretic mobility that were consistent with those reported in the literature [25]. Under the applied experimental conditions, the protein profile of both fresh (T_7_) and ripened (T_120_) cheese showed a major β-CN band and less intensive bands corresponding to αS1-CN and αS2-CN. In all samples, five low molecular weight peptides (from 25 kDa to 10 kDa) were all identified as proteolysis products. Dietary supplementation with zinc and iodine did not generate significant changes compared to the CG samples both at T_7_ and at T_120_, while selenium influenced the proteolytic process, partly protecting αS2-CN, as evidenced by the lack of significant differences in the proteolysis products corresponding to bands 2, 3, and 4 in SG samples. According to other studies, most of these peptides with molecular weight in the range 10–20 kDa were generated by rennet and plasmin following the degradation of αS-CN and β-CN [26,27]. Selenium has been reported to inhibit the expression of urokinase-type plasminogen activator (uPA), a serine protease, which can convert plasminogen to plasmin, which is capable of degrading extracellular matrix proteins and activating latent forms of MMPs [28]. The αS1 and β caseins did not show significant changes both in relation to the feeding strategy and in relation to the maturing time; this finding is consistent with what was previously reported in other studies and represents an added value if we consider the growing interest on β-casein micelles as a nano vehicle for solubility enhancement of natural compounds in the food industry [29].

Several studies have shown how changes in the diet of ruminants can be effective in inducing significant changes in the volatile profile of milk and dairy products [30]. Volatile compounds (VOCs) resulting from secondary proteolysis during ripening were identified in all the analyzed samples. Two volatile compounds, phenylacetaldehyde and 2-phenylethyl alcohol, derived from phenylalanine catabolism, while only the 3-methyl-1-butanol was identified as a derivative of leucine degradation. In both T_7_ and T_120_ samples, no significant variations were found in the phenylacetaldehyde content. As reported by McSweeney and Sousa [14], the phenylacetaldehyde metabolism in dairy products may occur by the nonenzymatic Strecker synthesis, through the degradation of phenylalanine, or by enzymatic transamination of phenylalanine as imide that is subsequently degraded to aldehyde. The presence of 2-phenylethyl alcohol is instead due to the phenylacetaldehyde reduction. In this case, the dietary supplementation with zinc, selenium, and iodine seems to have led to a significant reduction of this compound in fresh cheese samples, while in ripened samples, this finding was confirmed only for ZG samples. Aromatic compounds from phenylalanine have a relevant impact on the aroma of cheese. In particular, phenylethyl alcohol is reported to be one of the most odorous aromatic compounds, associated with a rose flower note [31]. With regard to leucine, this amino acid can undergo several biochemical processes in cheese during ripening. After an extracellular enzymatic degradation of casein by proteases, released from starter bacteria, leucine is converted to the corresponding α-keto acid (α-ketoisocaproic acid) by an intracellular transamination; then, such compound can be converted to α-hydroxy acids, aldehydes, or CoA-esters [32]. These leucine metabolites are not considered to be important for the development of cheese flavor, while the 3-methyl-1-butanol, derived from the hydrogenation of the corresponding aldehyde (3-methylbutanal), was reported to be responsible for alcoholic and fruity odors in Swiss-type cheese [33]. In this study, the only identified compound deriving from the leucine degradation was 3-methyl butanol, which tends to be synthesized in the various cheese samples with a comparable pattern both after 7 and 120 days of ripening. Specifically, no significant differences were observed between CG, ZG, and IG samples, while both at T_7_ and T_120_ of the SG, samples were characterized by a lower concentration of this compound. A similar behavior was also observed in a 30-days ripened caciotta cheese obtained from dairy cows fed organic selenium [34], as well as in a 90-days ripened Pecorino cheese deriving from ewes that received a dietary supplementation with organic zinc [35]. In addition to this, the dietary intake of organic zinc by dairy cows was even effective in inducing the reduction of 3-methyl butanol in a typical Italian soft cheese, the Giuncata cheese, after only 7 days storage at 4 °C [36]. Probably, the microelements administered in organic forms negatively influence the biochemical processes that lead to the formation of 3-methyl butanol, although the identification of the specific biochemical mechanisms needs further and more targeted investigations.

## 4. Materials and Methods

### 4.1. Experimental Design, Feeding Strategies, Cheesemaking, and Sampling

The experimental plan was performed according to Directive 2010/63/EU of the European Parliament (European Union, 2010) and Directive 86/609/EEC (European Economic Community, 1986), which deal with the protection of animals used for scientific purposes [37,38].

Thirty six healthy Friesian cows, homogeneous for age (41 ± 1.5 months) and lactation days (74 ± 12 days) have been used in this study. Animals, belonging to the same commercial farm, have been randomly divided into four groups of nine cows each. The control group (CG) was fed with a standard diet formulated taking into account the nutritional needs of cows in midlactation and guaranteeing each animal the daily requirement of each microelement, while the three experimental groups received the dietary supplementation with zinc (ZG), selenium (SG), and iodine (IG), respectively. The ZG received an additional total intake of about 100 mg of Zn. For the preparation of the rations, ZnO as a powder was used, and the dose management was performed according to Regulation (EC) No. 1831/2003 of the European Parliament and of the Council of 22 September 2003 (European Commission, 2003) on additives for use in animal nutrition [39]. With regards to SG, animals received a total daily supplementation of 0.47 mg of Se; current EU regulations have limited the use of Se supplementation to the overall content not exceeding 0.5 mg in complete feed daily administered. For the preparation of the ration organic Se as a crystalline powder was used that was incorporated into feed in the form of a premixture according to the recommendation reported in the Regulation No. 121/2014 of the European Commission concerning the authorization of l-selenomethionine as a feed additive for all animal species [40]. Finally, the IG animals were fed with a daily total iodine content of 4.5 mg/cow. For the dietary supplementation, potassium iodide (KI) as a powder was used and the total iodine was set not to exceed the maximum daily amount of 5 mg allowed by law (Reg. 1459/2005) [41].

The study had an overall duration of 70 days, characterized by 14 initial days of adaptation in which all the involved animals received the standard diet, followed by 56 days of dietary supplementation, in which animals of each group were housed in separate areas of free housing with an access to an identical feeding area in which each animal had an individual feeding bin with water freely available all throughout the study. All animals received about 23 kg/head/day of dry matter of total mixed rations (TMR) whose composition (Supplementary Table S2) was defined taking into account the parameters reported on the seventh edition of Nutrient Requirements of Dairy Cattle (2001) [42]. Samples of TMR were analyzed, according to AOAC methods (1990), for crude protein (CD; method 930.15), ether extract (EE; method 920.39), crude fiber (CF; method 962.09), and ash (method 942.05) [43]; detergent procedures reported by Van Soest et al. [44] were used for the determination of neutral detergent fiber (NDF) and acid detergent fiber (ADF). On the 70th day of the trial, individual raw milk samples were collected separately from each group and immediately analyzed for chemical composition. The remaining milk from each group was pooled and manipulated in the same way during the cheese-making process to obtain the Caciocavallo cheese, according to the protocol previously described [45]. In order to evaluate changes in the chemical composition and quality attributes due to ripening, sampling and analyses on Caciocavallo cheese were carried out after 7 days (T_7_) and 120 days (T_120_) from the cheese-making batches. Caciocavallo cheese is a dairy product that lends itself to being aged for quite long periods and on average it is consumed even after 6 months from cheesemaking. The analysis of the samples at the indicated times (T_7_ and T_120_) therefore allows us to compare the fresh product with a ripened product ready to be consumed. Samples, collected in triplicate from three different cheese-makings, were partly immediately analyzed and partly packed under vacuum and frozen at −20 °C until analysis.

### 4.2. Microelements Determination in Milk and Cheese

For the determination of the total amount of zinc in milk and cheese, samples were first mineralized by dry incineration, then subjected to atomic absorption spectrophotometry using an air/acetylene flame [46]. Selenium and iodine content was determined instead by inductively coupled plasma mass spectrometry (ICP-MS) by using an Agilent 7500ce (Agilent Technologies, Santa Clara, CA, USA) and following the procedures respectively reported by Gerber et al. and Fecher et al. [47,48].

### 4.3. Gelatin and Casein Zymography of Milk Samples

The evaluation of the activity of the zinc-dependent proteases in raw milk was performed through a zymographic approach. A total of 10 mL of each sample were placed in 15 mL tubes and centrifuged at 10,000 rpm for 20 min at 4 °C. This allowed us to obtain a separation in three distinct phases: an upper layer consisting of fat, a central serum fraction containing proteins, and a lower layer characterized by the cellular component and any interfering residues. The central phase was then carefully recovered and filtered through with 0.22 µm syringe filters before the total protein dosage that was calculated by the Bradford protein concentration assay [49].

Volumes of each sample corresponding to 10 µg of total proteins were diluted in a nonreducing sample buffer without heating, and resolved by 8% sodium dodecylsulphate polyacrylamide gel electrophoresis (SDS-PAGE) containing 0.2 mg/mL type B gelatin (Sigma Aldrich, Milan, Italy) [50]. The gels were then incubated for 45 min in a renaturation buffer (50 mM Tris-HCl pH 8.0, containing 2.5% Triton X-100) to remove SDS. Subsequently, incubation of 24 h in a developer buffer (50 mM Tris-HCl pH 8.0, containing 5 mM CaCl_2_, 200 mM NaCl, and 0.02% Brij 35) was performed to allow enzymes renaturation and activity. The gels were then stained in a 0.1% solution of Coomassie Blue R250 in 40% (*v*/*v*) methanol and 10% (*v*/*v*) acetic acid to allow the spot visualization.

The evaluation of caseinolytic activity was instead carried out by 10% SDS-PAGE with the addition of 0.3% of bovine casein (Sigma Aldrich, Milan, Italy). Additionally, in this case, volumes of each sample corresponding to 10 µg of total proteins were diluted in a nonreducing sample buffer without heating, before the electrophoretic resolving. After electrophoresis, gels were subjected to the same protocol reported for gelatin zymography, with the only variations regarding the final incubation period that was prolonged to 48 h.

The ImageJ software (version 1.44, National Institutes of Health, Bethesda, MD, USA) [51] was used to perform the quantitative analysis of visualized spots for both gelatin and casein zymography.

### 4.4. Caseins Extraction and Separation by Sodium Dodecyl Sulfate Polyacrylamide Gel Electrophoresis (SDS-PAGE)

Casein degradation in T_7_ and T_120_ cheeses was evaluated by sodium dodecyl sulfate polyacrylamide gel electrophoresis (SDS-PAGE). Each cheese sample (1 g) was dissolved in 20 mL 0.01 M Tris-Glicina pH 8.3 and 6 M Urea and homogenized for 2 min. The cheese extract was incubated for 2 h at 37 °C to induce the solubilization of casein fraction. The solution was then centrifugated for 15 min at 10,000 g (4 °C), and the supernatant was recovered and filtered through Whatman filter paper to remove fat and other insoluble solids.

Volumes of each sample, containing 10 µg of total extracted proteins, were diluted in a 2X sample buffer (62.5 mM Tris-HCl pH 6.8, 2% SDS, 10% glycerol, 5% β-mercaptoethanol, and traces of bromophenol blue), boiled for 3 min, and loaded onto a 15% polyacrylamide gel. The electrophoresis was performed in a mini-protean III dual slab cell (Bio-Rad Laboratories, Wartford, UK) at 30 mA constant amperage. Immediately after ending of the run, gels were placed at room temperature for 1 hr in a staining solution containing 40% methanol, 10% acetic acid, and 0.1% Coomassie Brilliant Blue G-250. Finally, the gels were destained by several washings in distilled water containing 40% methanol and 7% acetic acid. Densytometric analysis of displayed bands was performed by using ImageJ software [51].

### 4.5. Volatile Compounds Extraction and GC-MS Analysis

The extraction of volatile compounds from milk and cheese was performed by a solid-phase microextraction (SPME) and then analyzed using a gas-chromatograph coupled with a mass spectrophotometry (GC-MS) following the procedure previously described by Bennato et al. [52] with slight modifications. Grated cheese (4.5 g) was mixed with a saturated NaCl solution containing the internal standard, the 4-methyl-2-heptanone. The SPME was performed by exposing a 50/30 µm of divinylbenzene/carboxen/polydimethylsiloxane fiber (DVB/CAR/PDMS Supelco, Bellefonte, PA, USA) into the headspace of capped vials with a PTFE septum for 40 min at 50 °C in stirring conditions. Desorption of volatile compounds was obtained into the splitless injector of the GC system set at 250 °C for 1 min. The gas-chromatograph (Clarus 580; Perkin Elmer, Waltham, MA, USA) was coupled with a mass spectrometer (SQ8S; Perkin Elmer, Waltham, MA, USA) and equipped with a PE-ELITE-5MS 30 × 0.25 mm, 0.25 µm column (Perkin Elmer, Waltham, MA, USA). The oven temperature was set at 50 °C for 1 min, then was increased to 200 °C at 3 °C/min for 1 min, and to 250 °C at 15 °C/min, held for 15 min. The carrier gas was helium at 1 mL/min. Source and interface temperature were held at 250 °C. The mass detector operated in electronic impact mode (70 eV) and data were acquired in full scan mode (range 35–350 *m*/*z*, dwell time 0.2 s/scan). The volatile compounds were identified by comparing their mass spectra with those of the National Institute of Standards and Technology library (NIST, Gaithersburg, MD, USA) and comparing the eluting order with Kovats retention indexes. The isoamyl butyrate and isoamyl isobutyrate were identified comparing the mass spectra and the retention time of authentic standard compounds (Sigma Aldrich, St. Louis, MO, USA). Samples were analyzed in triplicate and quantification was carried out considering the relative peak area expressed as arbitrary unit (AU, target ion area × 10^−3^).

### 4.6. Statistical Analysis

The statistical analysis of data was carried out by using SAS software, version 9.2 (SAS Institute Inc., Cary, NY, USA). Data on volatile compounds have been processed with two-way ANOVA, considering diet, ripening time, and their interaction as fixed effects, according to the following statistical model: y_ijk_ = µ + D_i_ + T_j_ + D_i_ × T_j_ + e_ijk_, in which y_ijk_ = volatile compound, µ = population average, D_i_ = effect of dietary supplementation (CG, ZG, SG and IG), T_j_ = effect of ripening time (T_7_ vs. T_120_), D_i_ · T_j_ = interaction between dietary supplementation and ripening time, and e_ijk_ = error. Means separation was assessed by Tukey’s test and differences were declared significant at *p* < 0.05.

## 5. Conclusions

The results highlighted the possibility of fortification with zinc, selenium, and iodine in cheese through animal feeding. The increase in concentration of essential trace elements in dairy products, in addition to representing an advantage for the consumers health, undoubtedly influences the biochemical mechanisms that characterize cheese during aging, also contributing to the development of flavor. No variations were evidenced in the caseinolytic activity in raw milk, whereas in cheese, the electrophoretic analysis in denaturing and reducing conditions of cheese showed the ability of selenium to preserve αS1-CN from primary proteolysis during ripening. With regard to the evaluation of the proteolytic volatile profile, only three compounds, resulting from the degradation of phenylalanine and leucine, have been identified. In this case, further studies are needed to clearly understand the relationship between the dietary microelements supplementation and the characterization of the free amino acids pattern in fresh and ripened cheese, as well as the mechanisms involved in the production of proteolytic compounds.

## Figures and Tables

**Table 1:** Microelements quantification in milk samples obtained from lactating dairy cows fed control diet (CG) and control diet supplemented with zinc (ZG), selenium (SG), and iodine (IG).

Microelement	Milk Samples			
	CG	ZG	SG	IG
Zinc ^1^	4.18 ^a^ ± 0.37	5.76 ^b^ ± 0.41	3.98 ^a^ ± 0.33	4.04 ^a^ ± 0.40
Selenium ^1^	0.036 ^a^ ± 0.004	0.041 ^a^ ± 0.005	0.049 ^b^ ± 0.005	0.039 ^a^ ± 0.004
Iodine ^1^	0.12 ^a^ ± 0.03	0.11 ^a^ ± 0.02	0.10 ^a^ ± 0.02	0.17 ^b^ ± 0.02

^1^ Data are reported on a dry matter basis, as mean (mg·kg^−1^) ± standard deviation (S.D.). ^a,b^ Different letters in the same row indicate significant differences (*p* < 0.05).

**Table 2:** Microelements content in cheese samples obtained from lactating dairy cows fed control diet (CG) and control diet supplemented with different trace elements: zinc (ZG), selenium (SG), and iodine (IG).

Trace Element	Ripening Time ^1^							
	T_7_				T_120_			
	CG	ZG	SG	IG	CG	ZG	SG	IG
Zinc ^2^	41.34 ^a^ ± 2.03	52.61 ^b^ ± 2.37	42.77 ^a^ ± 2.19	40.77 ^a^ ± 1.98	43.21 ^a^ ± 2.41	54.74 ^b^ ± 2.39	41.82 ^a^ ± 3.09	42.91 ^a^ ± 2.93
Selenium ^2^	0.21 ^a^ ± 0.03	0.19 ^a^ ± 0.03	0.32 ^b^ ± 0.04	0.22 ^a^ ± 0.03	0.22 ^a^ ± 0.02	0.18 ^a^ ± 0.03	0.31 ^b^ ± 0.04	0.19 ^a^ ± 0.03
Iodine ^2^	0.21 ^a^ ± 0.03	0.24 ^a^ ± 0.03	0.19 ^a^ ± 0.03	0.31 ^b^ ± 0.04	0.20 ^a^ ± 0.03	0.22 ^a^ ± 0.03	0.18 ^a^ ± 0.02	0.29 ^b^ ± 0.04

^1^ 7 and 120 days of ripening (T_7_ and T_120_ respectively); ^2^ Data are reported in mg·kg^−1^ on a dry matter basis. ^a,b^ Different letters in the same row indicate significant differences (*p* < 0.05).

**Table 3:** Densitometric analysis of SDS-PAGE protein bands (Figure 3) in fresh (T_7_) and 120-days ripened (T_120_) cheese samples obtained from lactating dairy cows fed control diet (CG) and control diet supplemented with zinc (ZG), selenium (SG), and iodine (IG).

Protein	CG			ZG			SG			IG		
	T_7_	T_120_	*p*	T_7_	T_120_	*p*	T_7_	T_120_	*p*	T_7_	T_120_	*p*
α_S2_-CN	22.93 ± 2.07	26.59 ± 2.78	ns	20.98 ± 1.87	19.60 ± 1.79	ns	22.30 ± 2.13	21.02 ± 2.04	ns	23.80 ± 2.21	25.10 ± 2.33	ns
α_S1_-CN	33.16 ± 2.88	10.82 ± 0.85	**	29.66 ± 2.83	8.30 ± 0.78	**	31.82 ± 2.97	21.14 ± 1.99	**	29.98 ± 2.83	14.66 ± 1.42	**
β-CN	26.92 ± 2.54	27.94 ± 2.41	ns	30.57 ± 2.92	30.93 ± 2.82	ns	23.39 ± 2.18	27.21 ± 2.51	ns	31.33 ± 2.86	33.60 ± 3.11	ns
Band 1	5.04 ± 0.56	8.41 ± 0.86	*	5.19 ± 0.53	10.70 ± 0.96	**	5.48 ± 0.55	8.09 ± 0.78	*	4.10 ± 0.42	6.36 ± 0.61	*
Band 2	3.86 ± 0.44	6.36 ± 0.67	**	5.18 ± 0.52	8.76 ± 0.83	**	4.68 ± 0.47	5.96 ± 0.61	ns	3.09 ± 0.32	6.27 ± 0.62	**
Band 3	3.51 ± 0.39	6.77 ± 0.69	**	3.64 ± 0.38	8.20 ± 0.77	**	4.39 ± 0.45	4.27 ± 0.43	ns	2.76 ± 0.29	4.30 ± 0.42	*
Band 4	1.72 ± 0.23	6.70 ± 0.68	**	0.96 ± 0.11	6.77 ± 0.66	**	3.11 ± 0.33	3.99 ± 0.40	ns	2.10 ± 0.22	4.50 ± 0.44	**
Band 5	1.87 ± 0.21	6.41 ± 0.65	**	3.82 ± 0.39	6.74 ± 0.65	**	4.84 ± 0.50	8.32 ± 0.81	**	2.84 ± 0.28	5.21 ± 0.51	**

Data are reported as mean (%) ± S.D. of the total proteins found in the electrophoretic profile of each sample. α_S_-CN = α_S_-casein and β-CN = β-casein. Bands 1, 2, 3, 4, and 5 = fragments of protein degradation. * *p* < 0.05; ** *p* < 0.01; and ns = not significant.

**Table 4:** Proteolytic volatile compounds in fresh (T_7_) and 120-days ripened (T_120_) cheese obtained from lactating cows fed control diet (CG) and control diet supplemented with zinc (ZG), selenium (SG), and iodine (IG).

Volatile Compounds	T.I. ^2^	Ripening Time ^1^									
		T_7_					T_120_				
		CG	ZG	SG	IG	SEM	CG	ZG	SG	IG	SEM
Phenylalanine											
Phenylacetaldehyde	91	750	862	1083	480	227	3015	3658	5748	4334	1222
2-phenylethyl alcohol	91	6499 ^a^	3125 ^b^	1364 ^c^	3037 ^b^	399	9596 ^a^	6833 ^b^	7564 ^a^	14815 ^c^	2643
Leucine											
3-methyl-1-butanol	55	7481 ^a^	6526 ^a^	2697 ^b^	6091 ^a^	987	7648 ^a^	6327 ^a^	5921 ^b^	7597 ^a^	1179

^a,b,c^ Different letters in the same row indicate significant differences between groups (*p* < 0.05). ^1^ 7 and 120 days of ripening (T_7_ and T_120_ respectively); ^2^ Target ion. Data are reported as arbitrary units.

## Supplementary Materials

The following are available online at https://www.mdpi.com/1420-3049/25/9/2249/s1, Table S1: Chemical composition of fresh (T_7_) and 120-days ripened (T_120_) Caciocavallo cheese obtained from lactating dairy cows fed control diet (CG) and control diet supplemented with zinc (ZG), selenium (SG), and iodine (IG). Table S2: Ingredients and chemical composition of complete diets administered to lactating dairy cows belonging to control group (CG), zinc group (ZG), selenium group (SG), and iodine group (IG).
