# Supplementary material for: Proteolytic Volatile Profile and Electrophoretic Analysis of Casein Composition in Milk and Cheese Derived from Mironutrient-Fed Cows
Source: Molecules. 2020 May 10;25(9):2249. doi: 10.3390/molecules25092249 (PMC7249190; doi:10.3390/molecules25092249)
Supplement: Supplementary file 1 [file molecules-25-02249-s001.pdf]

molecules

Supplementary Materials

Table S1. Chemical composition of fresh (T_7) and 120-days ripened (T_{120}) Caciocavallo cheese obtained from lactating dairy cows fed control diet (CG) and control diet supplemented with zinc (ZG), selenium (SG) and iodine (IG).

Item	T7				T120			
	Diet				Diet			
	CG	ZG	SG	IG	CG	ZG	SG	IG
DM (%)	54.51 [A] ± 3.64	53.72 [A] ± 4.02	55.37 [A] ± 4.97	54.66 [A] ± 4.12	66.59 [B] ± 3.86	65.28 [B] ± 4.43	67.11 [B] ± 4.25	64.39 [B] ± 5.06
Fat (%) [1]	38.93 ± 3.08	40.45 ± 3.41	41.75 ± 3.62	41.15 ± 3.22	38.71 ± 3.30	37.49 ± 3.44	39.41 ± 3.84	38.92 ± 3.08
Protein (%) [1]	53.98 ± 4.18	51.22 ± 3.49	52.21 ± 2.95	50.07 ± 4.84	49.68 ± 3.92	48.60 ± 4.01	50.03 ± 4.16	47.93 ± 4.48
Ash (%) [1]	5.09 ± 0.41	5.33 ± 0.49	4.84 ± 0.48	5.76 ± 0.51	5.55 ± 0.51	5.79 ± 0.56	5.81 ± 0.57	5.91 ± 0.63

[1] Data are expressed on a dry matter (DM) basis as mean percentage (%) ± Standard Deviation. [A,B] Means with different superscripts are significantly different by ripening time ($p < 0.05$).

Table S2. Ingredients and chemical composition of complete diets administered to lactating dairy cows belonging to control group (CG), zinc group (ZG), selenium group (SG) and iodine group (IG).

**Ingredients**	
Corn silage, %	23.4
First cut, alfalfa hay, %	5.4
Corn meal, %	3.5
Soybean, meal, %	3.3
Fine bran, %	2.9
Barley, meal, %	2.0
$CaCO_3$, %	0.2
Vitamins and minerals, %	0.5
**Chemical composition**	
Dry Matter, %	57.03
Crude protein,[1] %	15.52
Ether extract,[1] %	2.84
Ash,[1] %	5.36
Neutral detergent fiber,[1] %	33.62
Acid detergent fiber,[1] %	20.96
Starch,[1] %	26.88
Zinc (ZG) [2]	95
Selenium (SG) [2]	0.47
Iodine (IG) [2]	4.5

[1] On a DM basis. [2] Daily total amount of each microelement reported as mg/animal.
